# Infected Cell Killing by HIV-1 Protease Promotes NF-κB Dependent HIV-1 Replication

**DOI:** 10.1371/journal.pone.0002112

**Published:** 2008-05-07

**Authors:** Gary D. Bren, Joe Whitman, Nathan Cummins, Brett Shepard, Stacey A. Rizza, Sergey A. Trushin, Andrew D. Badley

**Affiliations:** 1 Division of Infectious Diseases, Mayo Clinic, Rochester, Minnesota, United States of America; 2 Program in Translational Immunovirology and Biodefense, Mayo Clinic, Rochester, Minnesota, United States of America; Institute of Human Virology, United States of America

## Abstract

Acute HIV-1 infection of CD4 T cells often results in apoptotic death of infected cells, yet it is unclear what evolutionary advantage this offers to HIV-1. Given the independent observations that acute T cell HIV-1 infection results in (1) NF-κB activation, (2) caspase 8 dependent apoptosis, and that (3) caspase 8 directly activates NF-κB, we questioned whether these three events might be interrelated. We first show that HIV-1 infected T cell apoptosis, NF-κB activation, and caspase 8 cleavage by HIV-1 protease are coincident. Next we show that HIV-1 protease not only cleaves procaspase 8, producing Casp8p41, but also independently stimulates NF-κB activity. Finally, we demonstrate that the HIV protease cleavage of caspase 8 is necessary for optimal NF-κB activation and that the HIV-1 protease specific cleavage fragment Casp8p41 is sufficient to stimulate HIV-1 replication through NF-κB dependent HIV-LTR activation both in vitro as well as in cells from HIV infected donors. Consequently, the molecular events which promote death of HIV-1 infected T cells function dually to promote HIV-1 replication, thereby favoring the propagation and survival of HIV-1.

## Introduction

From a teleologic standpoint, it has been difficult to reconcile why a virally infected cell would undergo apoptosis, as it seems counter-intuitive that virus-initiated death of the host cell could offer a competitive advantage to that virus. Nonetheless, the majority of HIV-1 encoded proteins are capable of initiating death of both HIV-1 infected cells as well as uninfected bystander cells. The expression of active HIV-1 protease within the cytoplasm of infected cells, coupled with the degenerate substrate specificity of HIV-1 protease, allows for host cell proteins to be substrates of HIV-1 protease [1]. Cleavage of one of these host substrates by protease, procaspase 8, is a necessary event for death of infected T cells [2]. Following protease-mediated cleavage, a novel fragment of procaspase 8, called Casp8p41, is produced, which causes loss of mitochondrial transmembrane potential, release of cytochrome c, cleavage and activation of caspase 9 and 3 [2]. Casp8p41 is specific to HIV-1 protease initiated apoptosis, and is not produced during apoptosis induced by either death receptor signaling nor mitochondriotoxic stress [2]. The relevance of Casp8p41 to infected cell death is suggested by mutational inhibition of HIV-1 protease cleavage of procaspase 8, which inhibits HIV-1 killing of infected cells [3].

Recently, a non-apoptotic role for procaspase 8 has become recognized: NF-κB activation in response to antigen receptor, Fc receptor, or TLR2, 3, 4 ligation requires the presence of procaspase 8 [4], [5]. In response to these stimuli, procaspase 8 complexes with Iκκβ, resulting in phosphorylation and proteosomal degradation of Iκβα, followed by phosphorylation and nuclear translocation of p65 [4]. More recently, TRAF6 has been suggested to bind caspase 8, promoting the movement of this complex into lipid rafts [6]. The interaction of TRAF6 with caspase 8 is enhanced by caspase 8 processing [6], suggesting that cleavage of the caspase 8 zymogen enhances the ability of caspase 8 to activate NF-κB. Also, the structurally related cFLIP can initiate NF-κB activation via TRAF2 [7], in a manner that is enhanced by its prior cleavage by caspase 8 [8]. Since HIV-1 protease cleavage of procaspase 8 generates Casp8p41, we questioned whether Casp8p41 might also drive NF-κB activation and consequently HIV-LTR driven transcription.

## Materials and Methods

### Cell Culture

Jurkat (ATCC), J 1.1 (NIH AIDS Reference Reagent Program), and I 9.2 T-cells as well as primary human CD4+ cells were grown in RPMI 1640 supplemented with 10% fetal bovine serum and 2 mM Glutamine. Stably transfected I 9.2 cells were grown in RPMI 1640 with 10% FBS plus 500 ug/ml Geneticin (Invitrogen, Carlsbad, CA). 293T cells were cultured in DMEM plus 10% FBS and 2 mM Glutamine. Primary human peripheral blood lymphocytes were obtained following informed consent. This protocol was reviewed and approved by the Mayo Clinic and foundation institutional review board. HIV infections were performed using HIV IIIb (National Disease Research Interchange) using a high MOI of 2.5 mg/ml p24 in the infected supernatant.

### Plasmids and Transfection

Control of HIV-1 protease expression was accomplished by cloning the HIV-1 protease gene into the pTet On mammalian expression vector system (Invitrogen) and inducing expression with tetracycline as per manufacturer's recommendations.

The procaspase 8 cDNA was a gift from Dr. Marcus Peter. The HIV-1 protease resistant caspase 8 construct was made by mutating the Phenylalanine at amino acid 355 to Arginine and the Phenylalanine at 356 to Asparagine by site directed mutagenesis. The Casp8p41 construct was PCR amplified by designing a reverse primer with a stop codon at amino acid 356 (5′-TTAAAACACTTTGGGTTTTC-3′) coupled with a sense primer beginning at the initial ATG (5′-ATGGACTTCAGCAGAAAT-3′) and ligating into the pGEM-T Easy vector (Promega, Madison, WI) for subsequent cloning. The Casp8p41 ΔDED construct was PCR amplified using a sense primer beginning with the Methionine at amino acid 86 (5′-ATGGAAAGGGAACTT-3′) coupled with the Casp8p41 reverse primer and ligated into pGEM-T Easy (Promega). A dominant negative IκBα was created to block IKK activation of NF-κB by PCR generated mutagenesis of Serines 32 and 36 to Alanine [9]. For mammalian expression the constructs were cloned into either pEGFP or pcDNA3 (Invitrogen). All mutations and deletions were confirmed by DNA sequence analysis and tested for expression prior to experimental use.

The luciferase reporter constructs HIV-1 LTR Luc, HIV-1 LTR ΔTAR Luc, and HIV-1 LTR ΔKB Luc have been previously described [10]. The TK Renilla plasmid, purchased from Promega, was used as an internal control in all reporter plasmid transfections. Transfection efficacies of 30–40% are routinely achieved in these experiments as assessed by parallel transfections with expression vectors encoding fluorescent proteins. Results are expressed as luciferase per Renilla expression in order to normalize for variability between transfection efficiency and cell viability, between experimental groups, and between experiments.

Jurkat, I 9.2, and J 1.1 T-cells were transfected with 1 ug plasmid/10̂6 cells using an Electro Square Porator T820 (BTX, San Diego, CA) at 300 volts for 10 msec. Primary human CD4+ T-cells were harvested from normal or HIV-1 infected donors and purified using RosetteSep CD4+ Enrichment Cocktail (Stem Cell Technologies Inc.) and transfected using an Amaxa electroporator (Amaxa Inc., Koeln, Germany) with programmed routine U14 as per manufacturer's recommendations. 293T cells were transfected using Lipofectamine 2000 reagent (Invitrogen) as per manufacturer's instructions.

### Stable Cell Lines

The Jurkat T-derivative cell line, I 9.2, deficient in procaspase 8 protein expression, was stably transfected with expression vectors encoding for either green fluorescent protein (GFP), or procaspase 8 wild type conjugated to GFP (GFP-casp 8 WT) or the HIV-1 protease resistant construct of procaspase 8 conjugated to GFP (GFP-casp 8 RN). After transfection the cells were placed in media containing 800 ug/ml Geneticin and cultured for 14 days passing cells every 3 days with fresh media and Geneticin. The cells were then checked for GFP expression by fluorescent microscopy and for protein expression by western blotting. The transfected cells were maintained in media containing 500 ug/ml Geneticin. For electroporation experiments the Geneticin was removed 24 hours in advance.

### Nuclear Protein Extraction and Electromobility Shift Assay (EMSA)

293T cells were transfected with empty vector or plasmids coding for full length procaspase 8 or Casp8p41. After 6 hours at 37°C nuclear proteins were harvested as previously described [11]. 5 ug of nuclear protein was allowed to bind to a 32-P labeled, double stranded, oligonucleotide encompassing the NF-κB binding site of the HIV-1 LTR (5′-ACAAGGGACTTTCCGCTGGGGACTTTCCAGGG-3′) at room temperature in the presence or absence of antibodies specific for the NF-κB transcription factors p50 or p65 (Rel A) (Santa Cruz). The DNA-nuclear protein complexes were run on a 6% non-denaturing polyacrylamide gel at a constant voltage of 170volts. The gel was then dried onto filter paper and exposed to autoradiography film.

### HIV-1 p24 Antigen Assay

The chronically HIV-1 infected Jurkat T-cell clone J 1.1 was transfected with plasmids encoding for GFP, procaspase 8, or Casp8p41 with or without dominant negative IκBα (IκBα S 32/36 A). Culture supernatants were harvested 6 hours post transfection and assayed with the RETROtek HIV-1 p24 ELISA kit (ZeptoMetrix Corp., Buffalo, NY) for the presence of the HIV-1 antigen p24. Purified human CD4+ lymphocytes from normal and HIV-1 positive donors were transfected with plasmids encoding for procaspase 8, or Casp8p41, or GFP to monitor transfection efficiency. Culture supernatants were collected 18 hours post transfection and assayed as above.

Flow cytometry for Casp8p41 or p24 was performed on permealized cells using anti-Casp8p41 monoclonal antibody or anti-p24 FITC as previously described [3].

## Results

### HIV-1-induced activation of NF-κB is coincident with HIV-1-induced cell death

**Figure 1:**
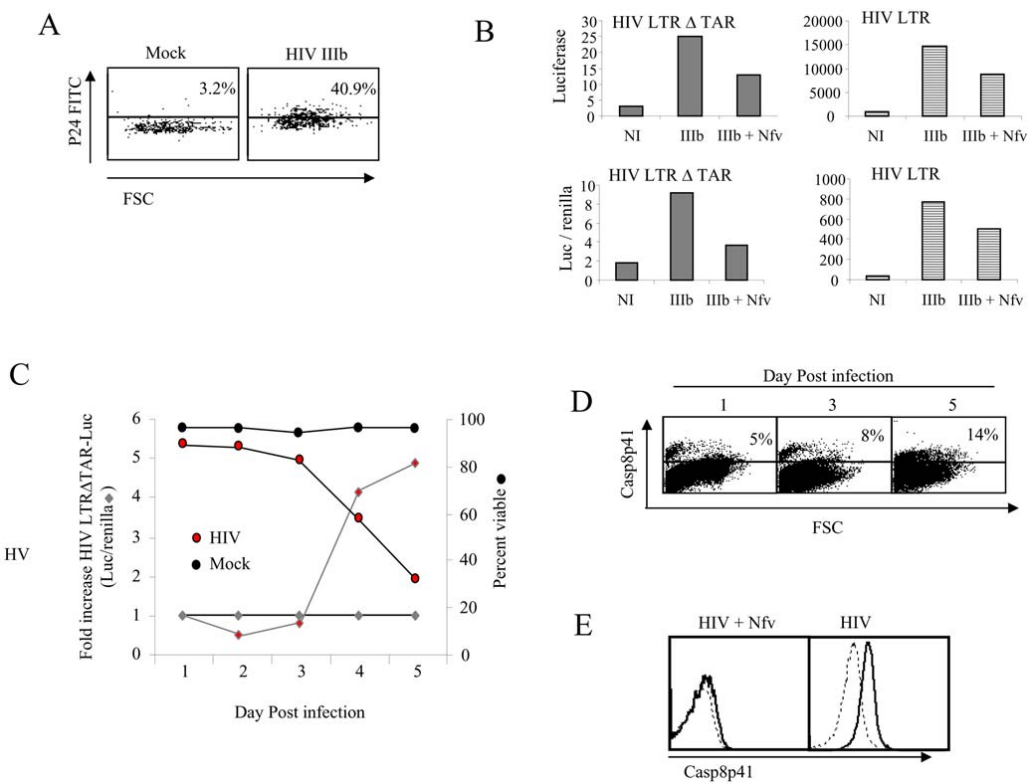
Jurkat T cell upregulate HIV LTR coincident with Casp8p41 expression. (A) Jurkat T cells were acutely infected with HIV IIIb or mock and three days post infection transfected with RFP. RFP positive cells were gated and p24 content analyzed by intracellular staining with p24 FITC. (B) HIV or mock infected Jurkat T cells were transfected with HIV LTR luciferase or HIV LTRΔTAR luciferase reporter constructs with or without Renilla cotransfection and luciferase measured in the presence or absence of the HIV protease inhibitor Nelfinavir (Nfv). (C) HIV IIIb or mock infected Jurkat T cells were analyzed daily for viability HIV LTRΔTAR luciferase/Renilla activity and (D) Casp8p41 content. (E) Casp8p41 was also measured in Day 3 HIV infected cells in the presence or absence of Nfv. All results representative of three or more experiments.

It is established that acute HIV-1 infection of CD4 T cells results in both NF-κB activation as well as apoptotic cell death [12]. However, the relative timing of these distinct processes remains undescribed. First we confirmed the ability of infected cultures to be efficiently transfected; mock or HIV infected cultures were transfected with RFP, and subsequently analyzed for p24 content. Of the RFP positive cells, 40.9% of cells in the HIV infected cultures were p24 positive, confirming that infected cells are efficiently transfected (Fig. 1A). Next we transfected luciferase reporter constructs into mock or HIV infected cultures, in the presence or absence of an HIV protease inhibitor, Nelfinavir (Nfv). Since a variety of signals can induce HIV LTR activity in infected cells, we compared HIV LTR with and without the TAR region deleted in order to control for the effect of TAT on NFκB. Both HIV LTR and HIV LTRΔTAR showed an increase in luciferase activity in Day 3 HIV IIIb infected cells that were diminished by Nfv (Fig. 1B). Luciferasae activity in the Nfv treated cells was however higher than mock infected cultures, likely due to the known effect of soluble factors, such as virion associated gp120 on NFκB mediated HIV replication [13]. Notably, the magnitude of luciferase induction in HIV infected cells is similar with respect to fold change in the HIV LTR compared to HIV LTRΔTAR, albeit the absolute magnitude of induction differed significantly. To control for potential differences in transfection efficiency as well as differences in expression between transfected cultures, Renilla was co-transfected and results expressed as luciferase per Renilla (Fig. 1B, bottom panels). As with luciferase expression alone, luciferase/Renilla expression showed similar effects due to HIV infection and Nfv treatment between HIV LTR and HIV LTRΔTAR. Therefore, in the ensuing experiments we used luciferase/Renilla. Next, we performed an acute HIV infection of Jurkat T cells, and analyzed daily viability and HIV LTRΔTAR activity normalized to Renilla. Mock infected cultures maintained viability of >95% throughout, and baseline levels of luc activity. HIV infected cultures reduced viability beginning on Day 3, and exhibited a reciprocal increase in luciferase (Fig. 1C). This increase in luciferase and decrease in viability occurred coincident to increases in Casp8p41 expression (Fig. 1D) raising the possibility that these events are related. Further HIV infection in the presence of Nfv which decreased luciferase activity (Fig. 1B) also reduced Casp8p41 expression (Fig. 1E).

### Expression of HIV-1 protease activates the HIV-1 LTR

**Figure 2:**
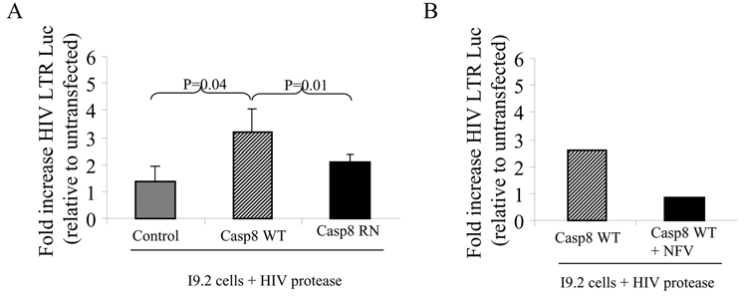
HIV protease upregulation of HIV LTR requires caspase 8. (A) The caspase 8 deficient 19.2 cell line was stably transfected with empty vector control, wild type caspase 8 (Casp8WT) or caspase 8 containing the HIV-1 protease resistant cleavage mutation RN at position 355/356 (Casp8RN), and then transfected with HIV-1 protease along with the HIV-LTR-Luc reporter construct. (B) 19.2 cells were transfected with Casp8WT in the presence or absence of Nelfinavir (Nfv) and then with HIV protease along with the HIV-LTR-Luc reporter construct. Results of three independent experiments expressed as fold increase relative to control, normalized to Renilla, +/− SD.

Since HIV-1 protease cleavage of procaspase 8 producing Casp8p41 is a necessary event for death of infected T cells [14], we questioned whether HIV-1 protease might also be responsible for the enhanced HIV-1 replication which coincides with infected cell death. For this, we co-expressed HIV-1 protease in cells containing an HIV-1 LTR luciferase reporter construct including the TAR region since TAT is absent in this model. In such experiments, HIV-1 protease induced 3 to 5 fold greater HIV-LTR activity than control (data not shown). Moreover, by using cells deficient in procaspase 8 (I9.2 cells) or the same cells engineered to express procaspase 8, we assessed the potential involvement of procaspase 8 in this process. I9.2 cells were stably transfected with vector control or wild type procaspase 8 (Casp8WT) or procaspase 8 with phenylalanines at positions 355 and 356, mutated to arginine and asparagine, respectively (Casp8RN), and expression of the transgene confirmed. We have previously determined that this Casp8RN mutant is resistant to HIV-1 protease cleavage [3]. When co-transfected with active HIV-1 protease in the presence of an HIV LTR luciferase reporter, the I9.2 cells stably expressing wild-type procaspase 8 showed an increase in transcriptional activity (absolute luciferase = 3565) compared to vector control, while expression of HIV-1 protease in cells containing the cleavage resistant Casp8RN mutant showed no increase in HIV-LTR transcriptional activity compared to vector control (Figure 2A). The increase in Luciferase induced by HIV protease in the Casp8WT cells was inhibited by Nelfinavir (Figure 2B). Therefore, optimal HIV-1 protease induced HIV LTR activation requires the presence of procaspase 8 and procaspase 8 must be in a form which is cleavable by HIV-1 protease.

### Casp8p41 activates the HIV-1 LTR in an NF-κB-dependent manner

**Figure 3:**
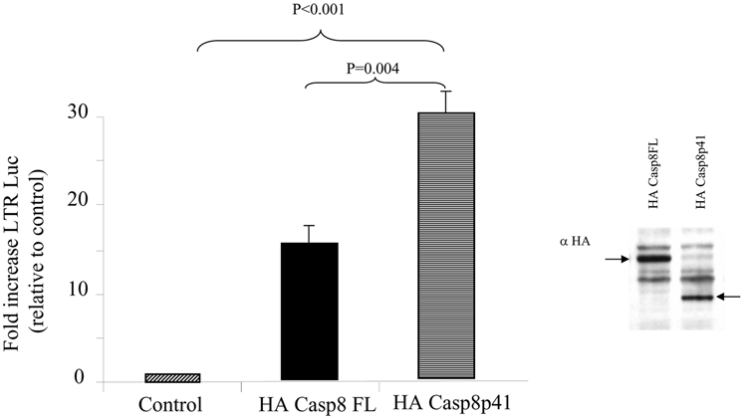
Casp8p41 upregulates HIV LTR. 293T cells were transfected with empty vector control, HA-Casp8FL or HA-Casp8p41, along with the HIV-LTR-Luc reporter construct. Transgene expression was confirmed by HA Western Blot. Results expressed as fold increase relative to control, normalized to Renilla. Results of three independent experiments expressed as fold increase relative to control, normalized to Renilla, +/− SD.

**Figure 4:**
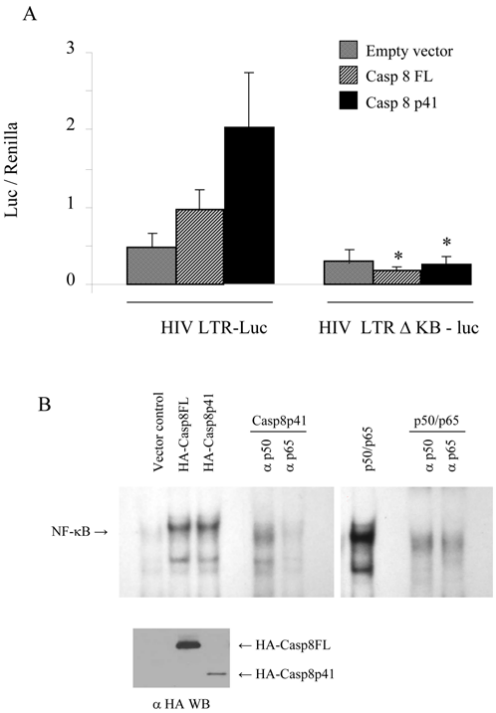
Casp8p41 upregulation of HIV LTR is NF-κB dependent. (A) 293T cells were transfected with empty vector control, Casp8FL or Casp8p41 along with HIV-LTR-Luc or with HIV-LTR-Luc with the KB site deleted (HIV-LTR-ΔKB-Luc) as indicated. Results of three independent experiments expressed as fold increase relative to control, normalized to Renilla, +/− SD. Results expressed as luciferase expression per Renilla. *p = <0.05 compared to HIV LTR. (B) 293T cells were transfected with empty vector control HA-Casp8FL or HA-Casp8p41 and analyzed by EMSA in the presence or absence of anti-p50 or anti-p65 antibodies. As a control for the antibodies EMSA's were performed in cells transfected with p50/p65 in the presence or absence of anti-p50 or p65 antibodies. The level of Casp8FL and Casp8p41 expression were determined in parallel.

Since HIV-1 protease activates the HIV-LTR in cells containing Casp8WT, but not the cleavage resistant Casp8RN mutant, the protease cleavage product of procaspase 8 may be directly involved in HIV-LTR activation. Therefore, we tested whether casp8p41 would independently activate HIV-LTR, and compared the effect of casp8p41 to full-length procaspase 8, the latter of which has been previously reported to stimulate NF-κB [5]. Full-length procaspase 8 (Casp8FL) or casp8p41 co-expression with an HIV LTR luciferase reporter plasmid, resulted in an increase in transcription that was greater in the case of casp8p41 than that of Casp8FL (Fig. 3). The Casp8p41 induced increase in transcriptional activity was due to NF-κB since the effect was abrogated in experiments using an HIV-LTR luciferase reporter construct with the NF-κB sites deleted (Figure 4A). The involvement of NF-κB was also confirmed by electromobility shift assay (EMSA), where expression of both full Casp8FL and Casp8p41 resulted in NF-κB up regulation (Figure 4B). The specificity of NF-κB response is confirmed by anti-p50 and anti-p65 antibodies. Comparing the NF-κB response with magnitude of transgene expression (Figure 4B bottom panel) confirms the enhanced NF-κB activation of Casp8p41 compared to Casp8FL, observed in previous experiments using reporter constructs.

### Casp8p41 transactivation of the HIV-1 LTR requires DED

**Figure 5:**
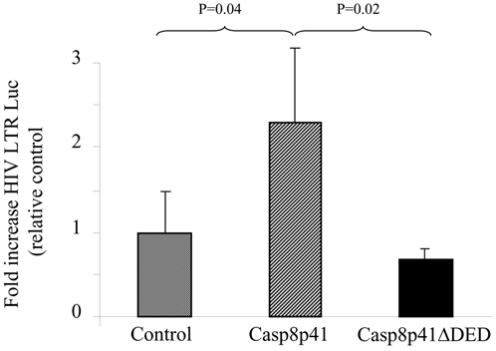
Casp8p41 upregulation of HIV LTR requires DED. 293 T cells were transfected with empty vector control, Casp8p41, or Casp8p41 with one DED deleted (Casp8p41 ΔDED), along with HIV-LTR-Luc, and luciferase measured. Results of three independent experiments expressed as fold increase relative to control, normalized to Renilla, +/− SD.

Previous studies have determined that the ability of c-FLIP to drive NF-κB activation is dependent upon the presence of two tandem DED motifs [8]. Consequently, we assessed whether the tandem DED motifs present within Casp8p41 were required for NF-κB activation. Casp8p41 or Casp8p41 containing a truncation of one DED (Casp8p41ΔDED) were coexpressed with the HIV LTR reporter construct. Expression of Casp8p41 induced high level HIV LTR transcriptional activity that was not present following expression of Casp8p41ΔDED (Figure 5).

### Casp8p41 induces HIV-1 replication

**Figure 6:**
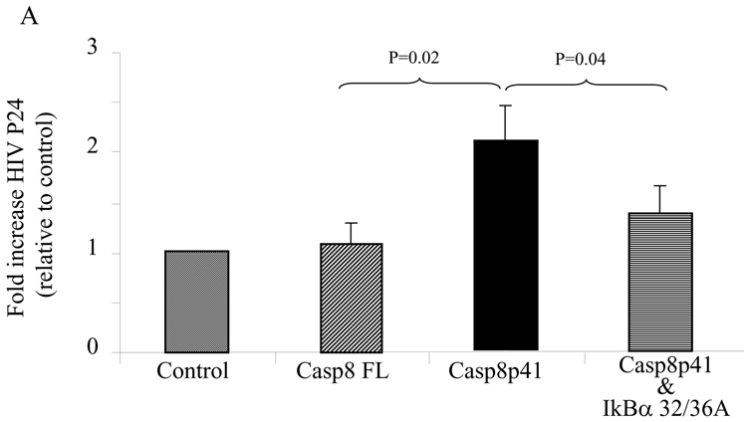
Casp8p41 upregulates HIV replication. J1.1 cells were transfected with empty vector control, full length caspase 8, Casp8p41, or Casp8p41 and dominant negative IκBα (IκBα 32/36A). P24 production in culture supernatants were determined. The limit of detection for the p24 assay is <5 pg/ml. Results of three independent experiments expressed as fold increase relative to control, normalized to Renilla, +/− SD.

In order for these effects of casp8p41 to be of relevance to HIV-1 pathogenesis, the ability of casp8p41 to activate NF-κB should translate into enhanced viral replication when infected cells express Casp8p41. We therefore tested whether or not Casp8p41 could increase the production of HIV-1 in an infected cell. To test this, we chose the Jurkat T cell line J1.1, which is chronically infected with HIV-1 [15]. In J1.1 cells, we expressed vector control, Casp8FL, Casp8p41, or Casp8p41 plus a dominant negative form of IκBα (IκBα 32/36A). The addition of IκBα 32/36Awas used to confirm that HIV-LTR activation via Casp8p41 occurred via the proposed interaction of caspase 8 with Iκκ resulting in IκBα phosphorylation and degradation. Six hours after transfection culture supernatants were assayed for the HIV-1 protein, p24, as a marker of HIV-1 replication. The expression of Casp8FL in J1.1 cells had minimal effect on production of p24. However, expression of casp8p41 resulted in an increase in p24 present in the culture supernatant, that was blocked by the dominant negative form of IκBα (Figure 6).

**Figure 7:**
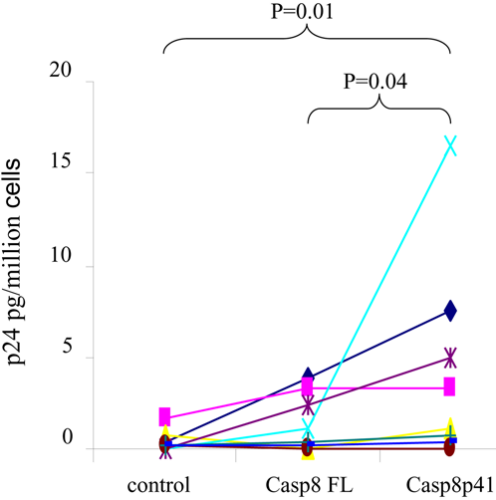
Primary CD4 T cells from HIV infected patients upregulate HIV replication in response to Casp8p41. Primary CD4 T cells from eight HIV positive or three HIV negative (data not shown) donors were transfected with empty vector control, Casp8FL, or Casp8p41, and p24 antigen production measured in triplicate. The limit of detection for the p24 assay is <5 pg/ml. Results presented represent means of replicates.

Finally, the effect of Casp8p41 on viral replication was assessed in primary CD4 T cells from HIV-1 infected or uninfected donors. Purified primary CD4 T cells were transfected with vector control, Casp8pFL or Casp8p41, and viral production by P24 antigen assessed. In 3 of 3 HIV negative donors, no p24 was detected by any treatment (data not shown). Eight HIV infected donors were assessed; all had CD4 counts >150, three patients had suppressed levels of viral replication, and the remaining five had viral loads between 100 and 73,400. Expression of Casp8FL resulted in an increase in p24 production relative to control plasmid, yet this effect was significantly less than the amount of p24 produced in response to expression of Casp8p41 (Figure 7). The level of p24 production following Casp8p41 expression did not correlate with plasma viremia, suggesting that level of integrated virus is not necessarily reflected by in plasma viral load, and that the level of integrated virus is which dictates the response to Casp8p41.

## Discussion

HIV-1 infection of lymphocytes results either in a productive infection with progeny virion production and death of the infected cell, or, less commonly, in a latent infection where the cell survives, yet the virus remains transcriptionally silent. The fact that acute HIV-1 infection of T cells results in the apoptotic death of those cells has been difficult to reconcile from a teleologic stand point, since most viruses, including HIV-1 infection of myeloid cells [16], invoke compensatory changes which favor viral persistence. Our current data suggest a unifying model wherein HIV-1 replication and consequent production of HIV-1 protease results in cleavage of procaspase 8 (which is expressed at high levels in activated T cells), causing two distinct but related events: mitochondrial depolarization and apoptosis, as well as NF-κB activation, and consequent increased production of progeny virions.

Since it has been previously reported that procaspase 8 can independently drive NF-κB activation, it is likely that procaspase 8 cleavage products should drive NF-κB as well. However, since most cell types contain procaspase 8, and not all such cells have high levels of NF-κB activity, it has been difficult to understand the relevance of full length procaspase 8 activation of NF-κB. Our data demonstrating that Casp8p41 more efficiently drives NF-κB and HIV-LTR transcriptional activity than does full length procaspase 8, suggest first, that caspase cleavage products are a more physiologic reason for NF-κB activation and second, that in those experiments where procaspase 8 activated NF-κB, perhaps the NF-κB activation was due to over expression of full length procaspase 8 which has previously reported to cause activation of caspase 8, consequent production of processing intermediates (e.g. p43). Our data also confirm that the structural components required for NF-κB activation are the tandem DED motifs and not the cysteine active site at position 360; furthermore, our data demonstrate that deletion of one DED is sufficient to abrogate the NF-κB response. Finally, procaspase 8 binding domains for TRAF6 at position 395–400 and 416-20, which are missing in Casp8p41, are not required for NF-κB activation.

Demonstration that Casp8p41 can directly initiate NF-κB dependent LTR activation, adds to our knowledge of what HIV specific factors drive HIV replication. First, HIV env binding to cells drives activation and NF-κB activation. Next, HIV TAT which is an early gene product potentially activates the LTR vial interaction with TAR. Later in the viral life cycle HIV Pr is produced which causes procaspase 8 cleavage resulting in production of Casp8p41. This in turn contributes to the death of infected cells [3], [14] and as we demonstrate in the current report, Casp8p41 also activates NF-κB dependent LTR activation. This effect is independent of TAT or env since reporter constructs deficient in TAR are increased following HIV infection and since Casp8p41 expression alone (without other HIV factors present) drives HIV LTR. Therefore, these data provide a conceptual model to explain the seeming paradox of why a virus would induce the death of a host cell; it does so in a manner which favors its own replication, such that progeny virions are produced which can infect other host cells.

Increased understanding of HIV-1 pathogenesis over the years has demonstrated the elegant ways that HIV-1 has adapted to use host metabolism to its advantage (e.g. utilizing LEDGF to facilitate integration) or ways that HIV-1 has adapted to over come cellular defenses (e.g. VIF and Apobec). In the current report we demonstrate that caspase 8 is necessary for optimum HIV-1 protease induced HIV-1 LTR activation (Figure 2) and that Casp8p41 expression is sufficient to initiate NF-κB dependent HIV-1 LTR activity (Figures 3 and 4), as well as HIV-1 replication (Figures 5, 6 and 7). The observation that HIV-1 protease cleavage of procaspase 8 causes cell death yet has also been adapted by HIV-1 to promote its own survival by enhancing NF-κB activation and consequent HIV-1 replication represents a novel case of how HIV-1 has adapted to overcome a natural host defense strategy.
